# Anaemia in Women: A Historical Example of Intersectionality in Health Care

**DOI:** 10.1111/1471-0528.70164

**Published:** 2026-01-22

**Authors:** Eric Jauniaux, Carolyn Paul

**Affiliations:** ^1^ EGA Institute for Women's Health, Faculty of Population Health Sciences University College London London UK; ^2^ Whittington Hospital London UK

**Keywords:** epidemiology: perinatal, health services research, maternal physiology, medical disorders in pregnancy

Anaemia affects women disproportionately due to its association with menstrual disorders and childbirth and often exacerbates the impact of obstetric complications, such as post‐partum haemorrhage. In December 2014, the World Health Organization (WHO) set a global nutrition target to achieve a 50% reduction of anaemia in women of childbearing age by 2025 (https://www.who.int/Anaemia in women and children). However, before the deadline was reached, it became obvious that progress towards this goal was not on track. The important role of social determinants of health was acknowledged, and it was recognised that a multisectoral approach would be required to address key barriers to reducing the prevalence of anaemia. In December 2023, a new framework was established with the later target date of 2030.

Accurate measurement of haemoglobin concentration is essential to provide a reliable estimate of the prevalence of anaemia in population‐based studies, and technical guidance, describing best practice, has been widely circulated. A landmark in the routine evaluation of full blood counts was the development of the automated Coulter Counter blood cell analyser by the American engineer Wallace Henry Coulter (1913–1998) in the early 1950s. This technique has enabled not only measurement of haematological indices but also evaluation of the impact of iron supplements during pregnancy, particularly on haemoglobin concentration (Taylor DJ and Lind T. *BJOG: An International Journal of Obstetrics & Gynaecology*, 1976;83:760–767).

The higher prevalence of anaemia among low‐income populations, an example of the intersection of health and poverty, has long been recognised. Molina et al., from Venezuela, identified that women from a poor economic background had lower blood levels of haemoglobin, serum iron levels and total iron binding capacity in samples collected during the first and third trimester of pregnancy, compared to women from a middle‐class background (*Journal of Obstetrics and Gynaecology of the British Commonwealth*, 1974;8:454–458).

Recently Bai et al. (*BJOG*, 2025; 132: 2097–2107), analysing the original data source from the Global Burden of Disease (GBD) study, highlighted the disproportionate burden of disease experienced by countries with lower socioeconomic conditions, particularly Western and Central sub‐Saharan Africa and South Asia, and the need for equitable resource allocation to these areas to accelerate progress if the new target is to be achieved.

Although the prevalence of inherited haematological disorders such as sickle cell disease and thalassaemia, and chronic exposure to parasitic disorders such as malaria are also high in Africa and South Asia, iron deficiency is still the main cause of anaemia in women of childbearing age. The treatment of iron deficiency in pregnancy has evolved over time, but medical research in the 1940s and 1950s highlighted the benefits of iron for both maternal health and foetal development (Davis LR and Jennison RF. *Journal of Obstetrics and Gynaecology of the British Empire* 1954;61:103–108) leading to the use of iron supplementation and infusions specifically for pregnant women (Figure 1).

**FIGURE 1:**
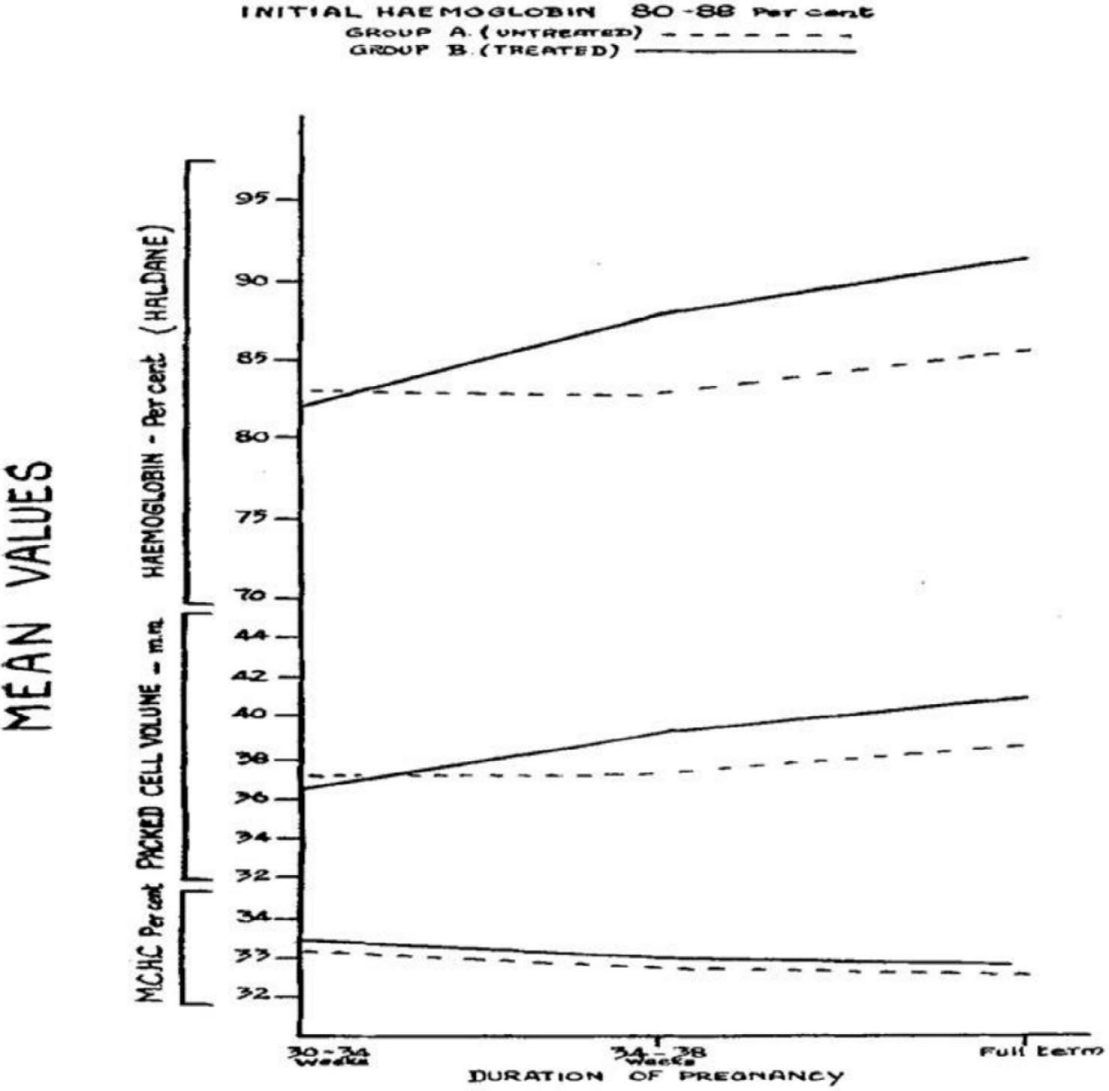
Changes in mean Hb values in untreated (A) and treated (B) groups at different gestational ages. From Davis LR and Jennison RF. *Journal of Obstetrics and Gynaecology of the British Empire* 1954;61:103–108.

Many governments prioritise iron supplementation in low‐resource countries. Still, challenges such as supply chain issues, access to health care, comprehensive dietary strategies and education remain obstacles to the implementation and effectiveness of these programs in many communities.

BJOG since 1902 Perspectives linked to BJOG‐25‐0142 (Global burden of anaemia among women of childbearing age: Temporal trends, inequalities and projections using the Global Burden of Disease 2021) for the special issue on Health Equity Research to Close Gaps in Women's and Reproductive Health.

## Author Contributions

Both authors conceived and wrote the first draft of the manuscript, critically revised the article content, and agreed on the final manuscript prior to submission.

## Funding

The authors have nothing to report.

## Conflicts of Interest

The authors declare no conflicts of interest.

## Linked Articles

Global Burden of Anaemia Among Women of Childbearing Age: Temporal Trends, Inequalities and Projections Using the Global Burden of Disease 2021, https://doi.org/10.1111/1471‐0528.18223.

